# Matching Donor and Recipient Size in Pediatric Heart Transplantation

**DOI:** 10.3389/ti.2022.10226

**Published:** 2022-02-07

**Authors:** Tajinder P. Singh, Steven D. Colan, Kimberlee Gauvreau

**Affiliations:** ^1^ Department of Cardiology, Boston Children’s Hospital, Boston, MA, United States; ^2^ Department of Pediatrics, Harvard Medical School, Boston, MA, United States; ^3^ Department of Biostatistics, Harvard School of Public Health, Boston, MA, United States

**Keywords:** heart transplant, children, pediatric, survival, donor selection, outcomes

## Abstract

Previous analyses in pediatric heart transplant (HT) recipients using weight or height have not found donor-recipient size-mismatch to be associated with post-transplant mortality. A recent study in 3,215 normal US children developed an equation for left ventricular (LV) mass using body surface area (BSA). We assessed whether donor-recipient size match using predicted LV mass (PLM) is associated with post-transplant in-hospital mortality or 1-year graft survival. We identified 4,717 children **<**18 yrs old who received primary HT in the US during 01/2000 to 03/2015 and divided them into five groups [10%, 10%, 60% (reference group), 10% and 10%, respectively] with increasing donor-recipient PLM ratio. In adjusted analysis, group 1 children (PLM ratio ≤.90) were at higher risk of post-transplant in-hospital mortality [Odds Ratio (OR) 1.55, 95% CI 1.04, 2.31]. This association of the most undersized donors with recipient in-hospital mortality was similar when donor-recipient weight ratio<.88 or BSA ratio<.92 (lowest decile) were used instead. There was no difference in 1-year graft survival among groups. Utilizing donors with donor-recipient PLM ratio ≤.90 is associated with higher risk of early post-transplant mortality in pediatric HT recipients. However, this metric is not superior to donor-recipient weight ratio or BSA ratio for assessing size match.

## Graphical Abstract

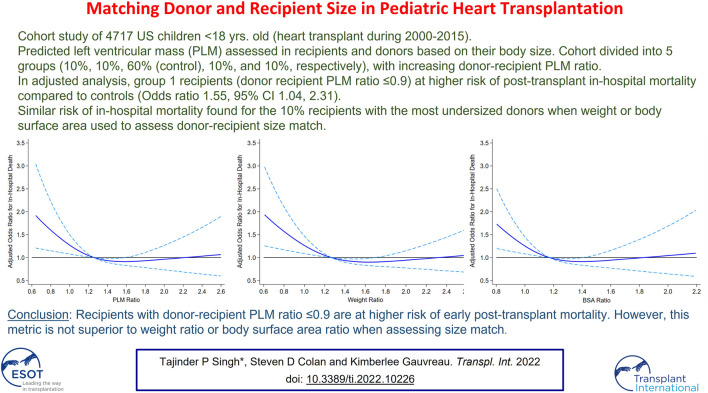

## Introduction

Transplant centers routinely provide a weight-range for an acceptable donor when listing a candidate for heart transplant (HT). This range is often 80%–200% of the recipient weight in children. Donor-recipient (DR) height match may also be considered when reviewing a donor offer. Size match using body measurements is essentially an attempt to match the donor and the recipient for their “normal” or “predicted” heart size to allow adequate mediastinal space for the donor heart and a donor heart that is able to support the recipient circulation after removal of the diseased heart. Previous analyses in pediatric HT recipients using weight or height to assess the effect of DR size match have shown either absent or only a marginal association of DR size-mismatch with recipient survival (1–3). This may be explained by a cautious selection of donors by pediatric HT community over the years such that a large enough sample of size-mismatched DR pairs to demonstrate an effect on graft outcomes does not exist. It may also be that body measurements such as weight or height may not be the best metrics to assess the association of DR size mismatch with outcomes.

Until recently, the practice of selecting donor size in adult HT candidates has been similar to the pediatric practice. After investigators of a population-based study (Multi Ethnic Study of Atherosclerosis) in the United States (US) performed cardiac magnetic resonance imaging (MRI) in healthy adults to develop normative equations for left ventricular (LV) and right ventricular (RV) mass using age, gender, height and weight (4, 5), several HT investigators have evaluated the role of predicted heart mass (predicted LV mass + predicted RV mass) as a potential metric for DR size match in adult HT. These analyses have found that recipients with hearts from undersized donors using this metric had significantly worse 1-year HT survival whereas size match assessed using weight, height, BSA or body mass index in the same patient population was not related (6). The superiority of assessing DR size match using predicted heart mass in adult HT recipients was also described in the 2019 annual report of the International Thoracic Registry (7). However, similar analyses were not performed in pediatric HT recipients because MRI-based values of RV or LV mass in normal children are limited to small studies and are not generalizable (3).

A recent Pediatric Heart Network study in 3,215 healthy, racially-diverse US children with adequate representation across the pediatric age range published an equation for left ventricular (LV) mass using body surface area (BSA) which can be used to estimate/predict LV mass for normal children with BSA of the HT recipient and the donor (8). Because LV mass is the dominant contributor to the heart mass after the neonatal period and potentially a surrogate for predicted heart mass (if value for RV mass is not available), we hypothesized that predicted LV mass (PLM) is a better metric for assessing DR size match compared to DR body measurements used in clinical practice and that DR size mismatch using PLM will be associated with short-term pediatric HT outcomes.

The specific aims of this study were 1) to assess the association of DR size match using predicted LV mass with post-transplant in-hospital mortality and 1-year graft survival in pediatric HT recipients and, 2), to compare its performance to the association of DR size-match using weight, height and BSA ratio with these outcomes in the same cohort.

## Materials and Methods

### Study Subjects

We identified all children <18 years old in the Organ Procurement and Transplant Network (OPTN) database who received first HT in the US between January 1st, 2000 and March 31st, 2015. Children who received heart re-transplant or multi-organ transplant were excluded. We also excluded recipients with missing weight or height for the recipient or the donor. The OPTN database includes baseline information at transplant and follow-up data in all recipients in the US submitted by transplant centers. These data are supplemented with death data from the social security master death file and are provided as de-identified data by the United Network for Organ Sharing to investigators. Post-transplant follow-up was available until March 31st, 2016, allowing 1 year of follow-up in all study children. The institutional review board determined that the study was not human subjects research as defined by US federal regulations.

### Study Design and Variables

This was a retrospective cohort study. Data were analyzed during March-December 2020. Two primary endpoints, post-transplant in-hospital mortality and graft loss during the first post-transplant year (time to death or re-transplant) were evaluated. The primary predictor was donor-recipient PLM ratio (=donor PLM divided by recipient PLM). This report follows the Strengthening the Reporting of Observational Studies in Epidemiology reporting guideline (9).

For all study subjects, BSA was calculated using the Haycock formula [BSA = 0.024265 x height (cm)^0.3964^ x weight (kg)^0.5378^] for both the recipient and the donor. This was used to generate PLM for all recipients and for donors up to 18 years old as follows (8):

Predicted LV mass = 53.02 × BSA^1.25^

For donors >18 years old (because pediatric equation is not validated), we calculated donor PLM using the MRI-derived adult equation (4):

PLM (>18 years) = a × Height^0.54^ (m) × Weight^0.61^ (kg)), where a = 6.82 for women, 8.25 for men.

Demographic and clinical variables were defined at transplant. Race/ethnicity was recorded as reported by center and analyzed as White (non-Hispanic White), Black (non-Hispanic Black), Hispanic or Other. Renal function was analyzed as estimated glomerular filtration rate (GFR, in ml/min/1.73 m^2^) using serum creatinine and the modified Schwartz equation (10). For children ≥1 year old, normal renal function was defined as GFR >60, moderate dysfunction as GFR 30–60, and severe dysfunction as GFR <30 or dialysis support. For infants <1 year old, normal renal function was defined as GFR >40, moderate dysfunction as GFR 20–40, and severe dysfunction as GFR <20 or dialysis support (11).

No subject had missing data for the variables age, gender, race/ethnicity, cardiac diagnosis, blood type, hemodynamic support (inotrope support, ventilator, type of mechanical support), health insurance (i.e., Medicaid), dialysis and the dates of transplant, death or re-transplant. For children with missing values of serum creatinine (2%) or bilirubin (7%), we used a multiple imputation technique to impute their GFR and serum bilirubin respectively using clinical variables at transplant and 10 imputations for each missing value (12).

### Statistical Analysis

Baseline characteristics are presented as median (Interquartile range, IQR) or number (percent). Study subjects were divided into five groups with increasing donor-recipient PLM ratio consisting of 10%, 10%, 60% (reference group), 10% and 10%, respectively of study subjects. This distribution was chosen to evaluate both ends of the size match spectrum (undersized and oversized donors including possible U-shaped relationship), with a reasonable sample size in exposure groups to detect the association of DR size mismatch with outcomes if present and to detect any trends with outcomes on either end, assuming the middle 60% would be the best matched group by size. The groups were compared for the distribution of baseline demographic and clinical (recipient and donor) variables as well as the distribution of DR weight-, height- and BSA ratio using chi-square tests or Kruskal-Wallis tests, as appropriate.

A multivariable logistic regression model using variables at HT and forward selection was developed for post-transplant in-hospital mortality retaining variables significant at the 0.10 level based on a likelihood ratio test; all variables in Table 1, other than DR weight-, height- and BSA ratio were considered. We decided a priori to adjust the model for the calendar year of HT irrespective of significance due to potential changes in clinical practices and recipients and improvement in early post-transplant survival over time (3). We then assessed the association of size match using donor-recipient PLM ratio adjusted for all factors in the model. We assessed the interaction of size match with model variables to determine a disproportionate effect, if any, on early post-transplant mortality. To compare the performance of PLM ratio with currently used metrics, we performed analyses using DR weight-, height- or BSA ratio (instead of PLM ratio) with post-transplant in-hospital mortality adjusted for all variables in the multivariable model. For each model, we used the middle 60% subjects for the corresponding variable as the reference group. We also evaluated adjusted risk of post-transplant in-hospital mortality with size match variables (PLM ratio, weight ratio and BSA ratio) assessed as continuous variables.

**TABLE 1:** Baseline characteristics of study children with increasing donor-recipient PLM ratio.

Variable	PLM Ratio0.55–0.90 (Group 1)	PLM Ratio0.91–1.00 (Group 2)	PLM Ratio1.01–1.60 (Group 3)	PLM Ratio1.61–1.83 (Group 4)	PLM Ratio1.84–3.40 (Group 5)	*p* value
	(*n* = 472)	(*n* = 472)	(*n* = 2,829)	(*n* = 472)	(*n* = 472)	
Age at transplant (years)						<.001
<1	149 (32%)	99 (21%)	759 (27%)	176 (37%)	214 (45%)	
1–10	202 (43%)	188 (40%)	1,023 (36%)	178 (38%)	192 (41%)	
11–17	121 (25%)	185 (39%)	1,047 (37%)	118 (25%)	66 (14%)	
Sex Male	255 (54%)	245 (52%)	1,527 (54%)	264 (56%)	289 (61%)	.032
Race/Ethnicity						.008
White	247 (52%)	266 (56%)	1,543 (55%)	257 (54%)	301 (64%)	
Black	104 (22%)	104 (22%)	595 (21%)	84 (18%)	73 (15%)	
Hispanic	92 (20%)	65 (14%)	494 (17%)	95 (20%)	68 (14%)	
Other	29 (6%)	37 (8%)	197 (7%)	36 (8%)	30 (6%)	
Blood type						.26
O	235 (50%)	207 (44%)	1,252 (44%)	217 (46%)	226 (48%)	
A	166 (35%)	188 (40%)	1,088 (38%)	179 (38%)	161 (34%)	
B	51 (11%)	54 (11%)	385 (14%)	56 (12%)	60 (13%)	
AB	20 (4%)	23 (5%)	104 (4%)	20 (4%)	25 (5%)	
Diagnosis						<.001
Dilated CMP	217 (46%)	214 (45%)	1,282 (45%)	179 (38%)	181 (38%)	
Non-dilated CMP	40 (8%)	55 (12%)	251 (9%)	31 (7%)	26 (6%)	
CHD repaired	161 (34%)	156 (33%)	979 (35%)	201 (43%)	183 (39%)	
CHD unrepaired	35 (7%)	31 (7%)	200 (7%)	42 (9%)	66 (14%)	
Other	19 (4%)	16 (3%)	117 (4%)	19 (4%)	16 (3%)	
Status at transplant						<.001
1A	379 (80%)	356 (75%)	2,251 (80%)	401 (85%)	409 (86%)	
1B	42 (9%)	56 (12%)	315 (11%)	47 (10%)	36 (8%)	
2	51 (11%)	60 (13%)	263 (9%)	24 (5%)	27 (6%)	
Ventilator	100 (21%)	66 (14%)	471 (17%)	107 (23%)	139 (29%)	<.001
Mechanical support						<.001
ECMO	30 (6%)	26 (6%)	153 (5%)	42 (9%)	46 (10%)	
BIVAD	25 (5%)	19 (4%)	164 (6%)	19 (4%)	19 (4%)	
LVAD	50 (11%)	51 (11%)	283 (10%)	29 (6%)	37 (8%)	
Inotropes	233 (49%)	240 (51%)	1,407 (50%)	268 (57%)	268 (57%)	.005
Bilirubin (mg/dl)	0.6 [0.3, 1.0]	0.7 [0.4, 1.1]	0.7 [0.4, 1.2]	0.6 [0.4, 1.3]	0.7 [0.4, 1.6]	<.001
Renal dysfunction						.042
Normal	409 (87%)	419 (89%)	2,439 (86%)	398 (84%)	383 (81%)	
Moderate	44 (9%)	38 (8%)	270 (10%)	50 (11%)	68 (14%)	
Severe	19 (4%)	15 (3%)	120 (4%)	24 (5%)	21 (4%)	
PRA (%)						.009
≤10	389 (82%)	375 (79%)	2,220 (78%)	387 (82%)	394 (83%)	
11–25	20 (4%)	38 (8%)	173 (6%)	20 (4%)	15 (3%)	
>25	63 (13%)	59 (13%)	436 (15%)	65 (14%)	63 (13%)	
Medicaid insurance	214 (45%)	194 (41%)	1,173 (41%)	204 (43%)	201 (43%)	.56
Year of transplant						.008
2000–2002	72 (15%)	77 (16%)	423 (15%)	65 (14%)	95 (20%)	
2003–2005	76 (16%)	63 (13%)	489 (17%)	98 (21%)	86 (18%)	
2006–2008	84 (18%)	91 (19%)	549 (19%)	102 (22%)	105 (22%)	
2009–2011	100 (21%)	103 (22%)	632 (22%)	94 (20%)	85 (18%)	
2012–2015	140 (30%)	138 (29%)	736 (26%)	113 (24%)	101 (21%)	
Donor age (years)						<.001
<1	197 (42%)	122 (26%)	648 (23%)	86 (18%)	52 (11%)	
1–10	179 (38%)	194 (41%)	1,046 (37%)	211 (45%)	267 (57%)	
11–17	79 (17%)	130 (28%)	656 (23%)	68 (14%)	57 (12%)	
≥18	17 (4%)	26 (6%)	479 (17%)	107 (23%)	96 (20%)	
Donor ischemic time (hours)						.039
<4	300 (64%)	295 (62%)	1826 (65%)	294 (62%)	260 (55%)	
≥4	152 (32%)	159 (34%)	893 (32%)	157 (33%)	187 (40%)	
Not reported	20 (4%)	18 (4%)	110 (4%)	21 (5%)	25 (5%)	
Donor-recipient weight ratio	0.83 [0.77, 0.88]	0.96 [0.92, 1.00]	1.27 [1.12, 1.46]	1.82 [1.69, 1.92]	2.24 [2.04, 2.50]	<.001
Donor-recipient height ratio	0.91 [0.85, 0.97]	0.97 [0.92, 1.02]	1.08 [1.01, 1.16]	1.23 [1.15, 1.32]	1.38 [1.27, 1.51]	<.001
Donor-recipient BSA ratio	0.87 [0.84, 0.90]	0.97 [0.95, 0.99]	1.17 [1.08, 1.29]	1.51 [1.47, 1.56]	1.75 [1.66, 1.89]	<.001
Male recipient/Female donor	103 (22%)	95 (20%)	595 (21%)	108 (23%)	136 (29%)	.004

Data are expressed as number (%) or median (interquartile range), PLM, predicted left ventricular mass; CMP, cardiomyopathy; CHD, congenital heart disease; ECMO, extracorporeal membrane oxygenation; BIVAD, biventricular assist device; LVAD, left ventricular assist device; PRA, panel reactive antibody; BSA, body surface area.

Kaplan Meier curves and log rank test were used to compare cumulative 1st year post-HT graft loss (death or re-HT) among the five groups. Multivariable Cox models were built to assess the association of size match using different metrics for 1 year graft survival, adjusted for baseline characteristics and year of transplant. For each model, the middle 60% subjects for the specific metric were used as the reference group.

We performed a sensitivity analysis by repeating/limiting all multivariable analyses only in recipients who received a heart from a donor up to 18 years old so that both the recipient and the donor PLM were derived using the PHN equation.

Data were analyzed using SAS version 9.4 (SAS Institute, Inc., Cary, NC) and Stata version 15 (StataCorp, College Station, TX). All statistical tests were two-sided and a *p* < 0.05 defined statistical significance. The authors had full access to the data and take responsibility for its integrity. All authors have read and agree to the manuscript as written.

## Results

During the 15 year study period, 4,797 children <18 years old underwent primary HT in the US. Of these, 23 received a multi-organ transplant and 57 had missing weight or height for the recipient or the donor and were excluded. The remaining 4,717 children in whom PLM could be estimated for both the recipient and the donor formed the study cohort. Of these, 30% were infants <1 year old, 55% were male, 52% had cardiomyopathy, 44% had congenital heart disease and 21% were on a mechanical support (6% on extracorporeal membrane oxygenation, 5% on biventricular assist device and 10% on left ventricular assist device) at transplant. Overall, 85% of these recipients received a heart from a pediatric donor <18 years old, the remaining being adult donors.

**FIGURE 1:**
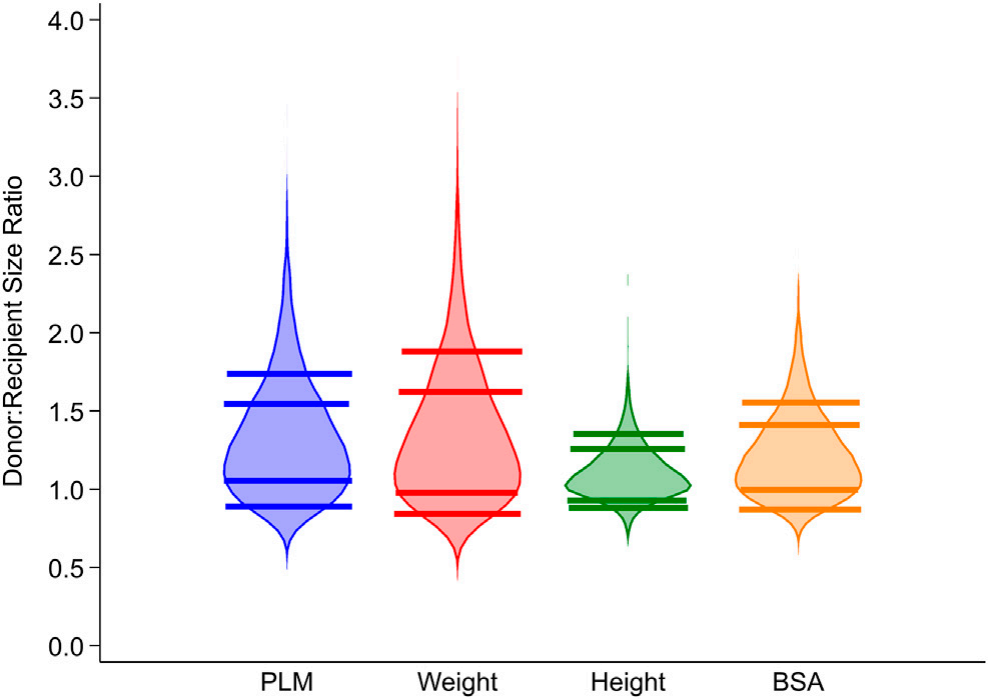
Distribution of donor-recipient PLM ratio, weight ratio, height ratio and BSA ratio in the study cohort. PLM ratio, weight ratio, height ratio and BSA ratio were used to divide the study cohort into five groups representing 10%, 10%, 60% (reference group), 10% and 10% of subjects with increasing value of the ratio. PLM, predicted left ventricular mass; BSA, body surface area.

Donor-recipient PLM ratio ranged from 0.55 to 3.40 in the study cohort and was 0.55–0.90 in group 1 (most undersized donors), 0.91–1.00 in group 2, 1.01–1.60 in group 3 (reference group), 1.61–1.83 in group 4 and 1.84–3.40 in group 5 (most oversized donors), respectively. The distribution of baseline recipient and donor characteristics among the five groups with increasing donor-recipient PLM ratio is illustrated in Table 1. As expected, recipients with higher donor-recipient PLM ratio had higher DR weight ratio, higher DR height ratio and higher BSA ratio (*p* for trend<.001 for all, Table 1). Figure 1 illustrates violin plots with the distribution of study cohort into five groups (10%, 10%, 60%, 10% and 10%) using donor-recipient PLM, weight, height and BSA ratio, respectively.

### Post-Transplant In-Hospital Mortality

Overall, 283 (6%) children died prior to hospital discharge. In-hospital mortality was 8.3%, 4.9%, 5.5%, 7.0% and 6.8%, respectively in PLM Groups 1–5 (*p* = .10). In multivariable analysis, recipient age, cardiac diagnosis, ventilator or mechanical support, renal dysfunction, hepatic dysfunction and donor ischemic time were all significantly associated with in-hospital mortality (Table 2). In adjusted analysis (adjusted for factors in Table 2), HT recipients with the lowest donor-recipient PLM ratio (group 1, PLM ratio ≤.9) were at a significantly higher risk of in-hospital mortality [Odds Ratio (OR) 1.55, 95% CI 1.04, 2.32, *p* = .03] compared to the reference group (PLM group 3) whereas HT recipients in PLM group 2 (OR 1.01, 95% CI 0.62, 1.64), group 4 (OR 0.95, 95% CI 0.62, 1.47) or group 5 (OR 0.78, 95% CI 0.50, 1.20) were not at higher risk of in-hospital mortality. There was no significant interaction of PLM group 1 with any risk factor in the multivariable model.

**TABLE 2:** Multivariable model for post-transplant in-hospital mortality.

	Odds ratio	95% confidence interval	*p* value
Age at transplant <1 Year	1.99	1.48, 2.67	<.001
Diagnosis (vs. Dilated CMP)			<.001
Non-dilated CMP	1.86	0.99, 3.50	
CHD repaired	3.89	2.75, 5.50	
CHD unrepaired	1.56	0.90, 2.69	
Other	1.91	0.94, 3.87	
Ventilator	1.84	1.36, 2.50	<.001
Mechanical support (vs. none)			<.001
ECMO	3.30	2.30, 4.74	
BIVAD	2.23	1.25, 3.98	
LVAD	1.08	0.57, 2.04	
Bilirubin (vs. < 0.6 mg/dl)			<.001
0.6–1.9	1.55	1.12, 2.14	
≥2.0	2.26	1.55, 3.30	
Renal dysfunction (vs. normal)			<.001
Mild-moderate	2.06	1.45, 2.94	
Severe	3.88	2.59, 5.80	
Donor ischemic time (vs. < 4 h)			.002
≥4	1.59	1.21, 2.09	
Not reported	1.67	0.91, 3.07	
Male recipient/Female donor	0.74	0.54, 1.02	.068
Year of transplant (vs. 2000–2002)			
2003–2005	1.04	0.68, 1.58	
2006–2008	0.82	0.53, 1.27	
2009–2011	0.81	0.52, 1.27	
2012–2015	0.86	0.56, 1.31	

CMP, cardiomyopathy; CHD, congenital heart disease; ECMO, extracorporeal membrane oxygenation; BIVAD, biventricular assist device; LVAD, left ventricular assist device.

There was no difference in the distribution of causes of in-hospital mortality among PLM groups 1–5. There was also no difference among groups in the proportion of children who developed severe primary graft dysfunction (6%, 4%, 4%, 7%, 6%, respectively, *p* = .11), defined as initiation of extra-corporeal membrane oxygenation support within 2 days following transplant (13, 14). However, the association of PLM group 1 with in-hospital mortality was weaker (adjusted OR 1.45, 95% CI 0.95, 2.22) when primary graft dysfunction (yes/no) variable was added to the multivariable model.

There was a borderline increased risk of in-hospital mortality in adjusted analysis in recipients in the lowest decile of DR weight ratio defined as <0.88 (OR 1.49, 95% CI 0.99, 2.25, *p* = .05, Supplementary Table S1) whereas recipients in the lowest decile of DR height ratio were not at increased risk (OR 1.15, 95% CI 0.74, 1.77, *p* = .54, Supplementary Table S1). Using BSA ratio for DR size match demonstrated a significantly increased risk of in-hospital mortality among recipients in the lowest decile, defined as <0.92 (OR 1.53, 95% CI 1.02, 2.30, *p* = .04). The area under the receiver operating characteristic curve for the multivariable models for in-hospital mortality was identical (c statistic = 0.81) whether donor-recipient PLM ratio, weight ratio or BSA ratio was used in the multivariable model.

**FIGURE 2:**
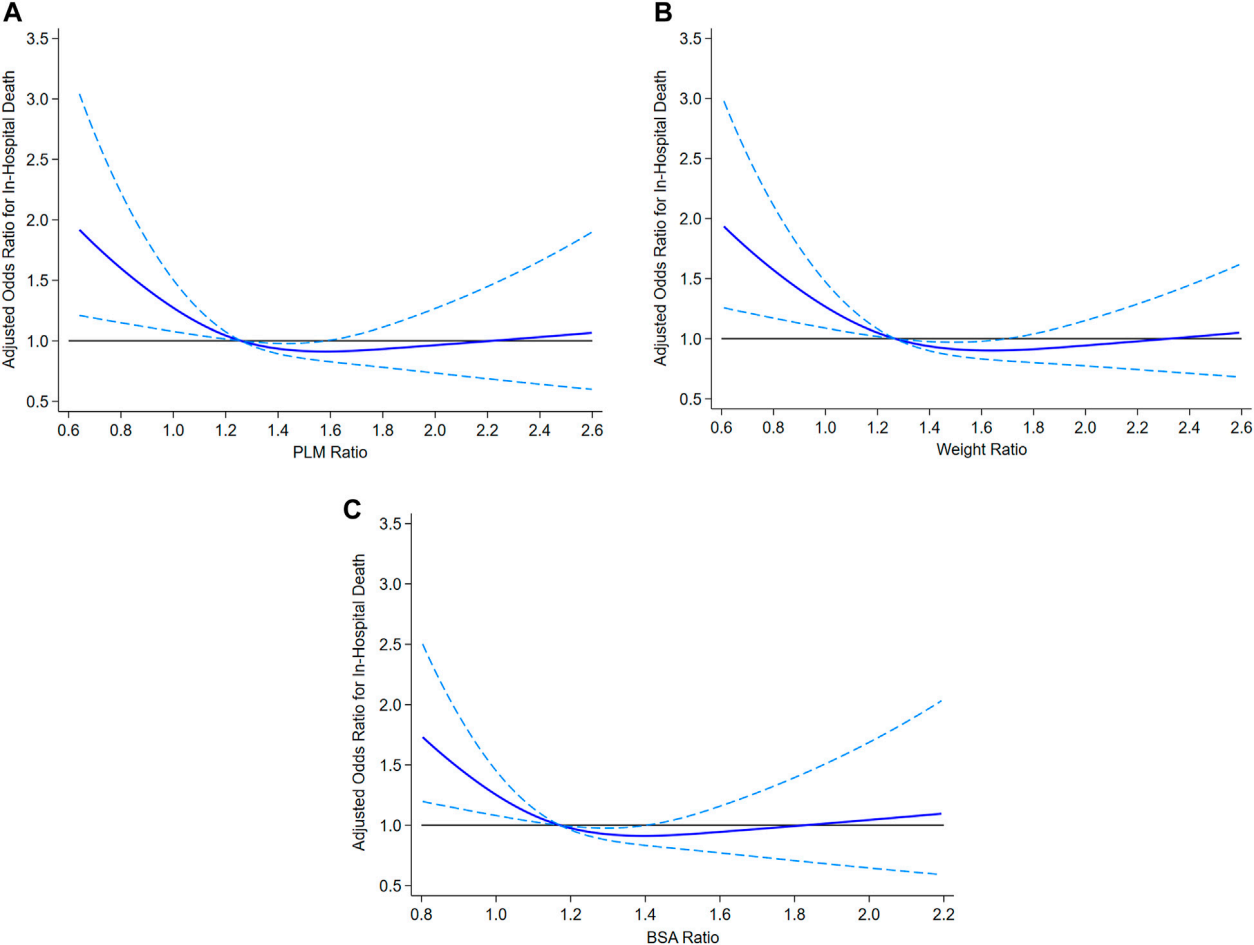
Association of post-transplant in-hospital mortality with donor-recipient size match assessed as PLM ratio (2A), weight ratio (2B) and BSA ratio (2C). PLM, predicted left ventricular mass; BSA, body surface area.

Figure 2 illustrates the association of post-transplant in-hospital mortality with DR size match when donor-recipient PLM ratio (2A), weight ratio (2B), and BSA ratio (2C) were assessed as continuous variables.

The donor age was 18 years or younger for 3992 HT recipients in the study and therefore the PHN equation for PLM was applicable for both the donor and the recipient. Among these recipients, those with the lowest donor-recipient size ratio were at higher risk of post-transplant in-hospital mortality in adjusted analysis whether donor-recipient PLM ratio (OR 1.55, 95% CI 1.03, 2.35), weight ratio (OR 1.59, 95% CI 1.04, 2.45) or BSA ratio (OR 1.62, 95% CI 1.06, 2.48) were used to define the most undersized decile of donors.

### Post-Transplant 1-Year Graft Survival

**FIGURE 3:**
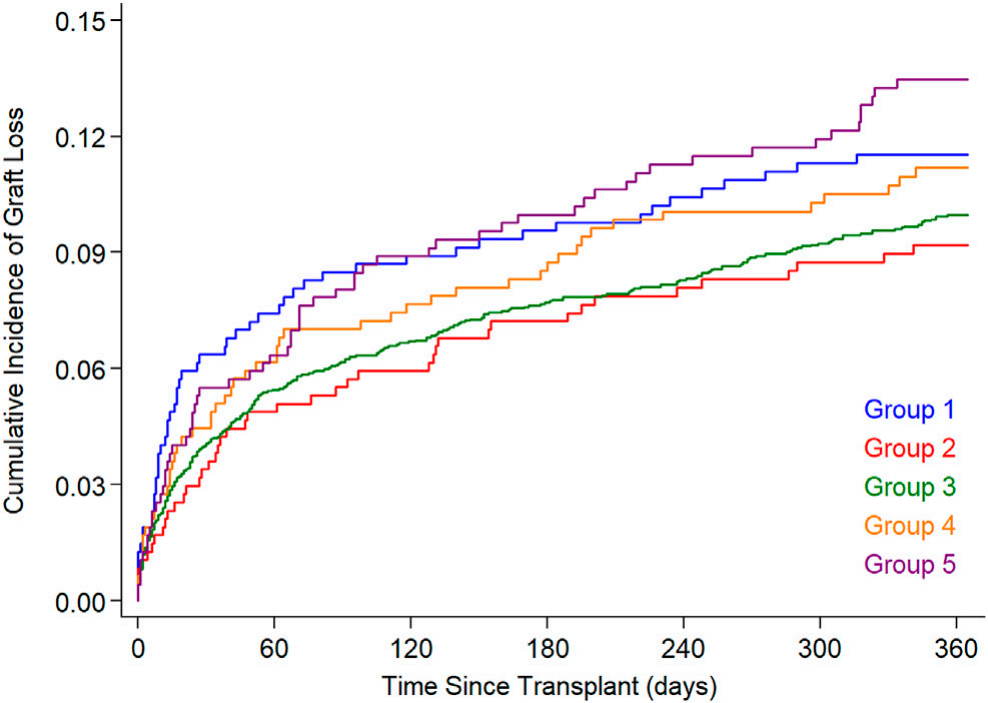
Cumulative graft loss (death or re-transplant) in the 5 PLM groups. The PLM groups 1–5 represent 10%, 10%, 60% (reference group), 10% and 10% of study subjects with increasing PLM ratio. PLM, predicted left ventricular mass.

Figure 3 illustrates cumulative 1 year graft loss among HT recipients stratified by donor-recipient PLM ratio. Graft loss during the first post-transplant year occurred in 11.4%, 9.1%, 9.9%, 11.0%, and 13.4% in Groups 1–5. The difference among groups was not statistically significant (*p* = .14, log rank test).

In a multivariable Cox model, risk factors associated with graft loss during the first post-transplant year included recipient age, cardiac diagnosis, black race, hemodynamic support at transplant, renal or hepatic dysfunction and donor ischemic time (Table 3). PRA was not associated with survival. In analysis adjusted for factors in Table 3, HT with either undersized donors (PLM group 1, hazard ratio [HR] 1.20, 95% CI 0.89, 1.61; PLM group 2, HR 1.03, 95% CI 0.74, 1.42) or with oversized donors (PLM group 4, HR 0.91, 95% CI 0.67, 1.22; PLM group 5, HR 1.00, 95% CI 0.75, 1.32) was not associated with 1 year graft loss. Similarly, there was no association of donor-recipient size mismatch with graft loss during the first year when the DR size match groups were based on the distribution of DR weight-, height or BSA ratio (Supplementary Table S2). There was no association of size mismatch with 1 year graft loss when the analysis was limited to donors up to 18 years old.

**TABLE 3:** Multivariable cox model for graft loss within 1 Year of heart transplant.

	Hazard ratio	95% confidence interval	*p* value
Age at transplant <1 year	1.58	1.29, 1.94	<.001
Race/Ethnicity (vs. White)			.003
Black	1.33	1.06, 1.67	
Hispanic	0.77	0.58, 1.02	
Other	1.33	0.94, 1.90	
Diagnosis (vs. Dilated CMP)			<.001
Non-dilated CMP	1.85	1.24, 2.76	
CHD repaired	2.92	2.31, 3.68	
CHD unrepaired	1.57	1.08, 2.27	
Other	1.40	0.82, 2.36	
Ventilator	1.56	1.26, 1.94	<.001
Mechanical support (vs. none)			<.001
ECMO	2.58	2.00, 3.32	
BIVAD	1.82	1.23, 2.71	
LVAD	0.98	0.63, 1.52	
Bilirubin (mg/dl) (vs. < 0.6)			<0.001
0.6–1.9	1.23	0.99, 1.53	
≥2.0	1.59	1.23, 2.05	
Renal dysfunction (vs. none)			<.001
Mild-moderate	1.59	1.23, 2.04	
Severe	2.72	2.07, 3.59	
Donor ischemic time (hours) (vs. < 4)			.001
≥4	1.29	1.07, 1.55	
Not reported	1.24	0.81, 1.90	
Male recipient/Female donor	0.72	0.58, 0.90	.003
Year of transplant (vs. 2000–2002)			<.001
2003–2005	1.00	0.76, 1.33	
2006–2008	0.90	0.67, 1.19	
2009–2011	0.75	0.55, 1.01	
2012–2015	0.64	0.47, 0.86	

CMP, cardiomyopathy; CHD, congenital heart disease; ECMO, extracorporeal membrane oxygenation; BIVAD, biventricular assist device; LVAD, left ventricular assist device.

## Discussion

A longstanding wisdom when evaluating a donor for HT is to avoid undersized donors due to the risk of primary graft failure in the recipient. In this study of US children who received primary HT in the US during a 15 year period, we calculated donor and recipient PLM using a recently described equation in normal US children. We found that 10% of HT recipients received a heart with donor-recipient PLM ratio of ≤.90. These children were at 55% higher risk of post-transplant in-hospital mortality compared to the reference group in adjusted analysis. When size match was assessed with PLM ratio as a continuous variable, the adjusted risk of in-hospital mortality was higher the more undersized the donor heart. Recipients who received oversized hearts were not at increased risk. There was no association of DR size mismatch with 1 year graft survival suggesting that the risk associated with using hearts from undersized donors is short-term. The association of undersized donors with in-hospital mortality was also demonstrable to a comparable degree when size match was assessed using DR weight ratio or donor-recipient BSA ratio. These findings are different from analyses in adult HT recipients where use of predicted heart mass formula to assess DR size match is superior to using body measurements. Considering the lack of superiority of PLM ratio and the simplicity in using DR weight ratio or BSA ratio when evaluating size match, it is difficult to justify a routine use of donor-recipient PLM ratio when evaluating donors. DR height ratio was not associated with post-transplant in-hospital mortality or 1 year graft loss.

We were inspired to ask the study question after several studies in adult heart transplantation (6, 15) and the 2019 annual report of the International Thoracic Registry in adult HT recipients showed that DR predicted heart mass ratio was the optimal metric for assessing DR size match by being associated with 1 year post-transplant survival whereas DR weight-, height- or BSA ratio were not (7). Prior to these reports, DR weight ratio was the most common metric for assessing size match in adult HT candidates (16). The ability to estimate predicted heart mass in adults followed publications of normative equations for LV and RV mass using gender, height and weight based on cardiac MRI data in a multi-ethnic population-based study in the US (4, 5). These equations have not been validated in children and MRI-based values of RV or LV mass in normal children are limited to small studies (17). Echocardiography is limited in its ability to image RV due to its proximity to sternum, its geometry and a thin RV free wall. LV mass measurements have however been routinely performed in clinical practice using echocardiography (18). LV mass is the dominant contributor to the heart mass after the first 4–6 weeks of life. This is supported by an MRI study in 50 healthy children where the BSA-based regression equations showed the mean LV mass to be > 3 times the RV mass during childhood (17). This is similar to adults where applying the MRI-derived equations to a few real life examples shows that LV mass contributes 75%–80% to the predicted heart mass (4, 5). Lacking an equation for predicted RV mass in children, we reasoned that LV mass would contribute about the same proportion to total heart mass in most children making predicted LV mass a reasonable surrogate for predicted heart mass and designed the current study as we did.

Previous analyses in children using weight or height have shown absent or marginal association of DR size-mismatch with recipient survival. Tang et al analyzed 3048 US pediatric HT recipients during 1994–2008 for DR size match using weight (1). There were 204 (6.7%) recipients with donor weight <80% of the recipient weight. They found no effect on post-transplant survival when the donor weight was 60%–80% of the recipient weight but reported lower 30 day survival in infant recipients with donor weight <60% of the recipient weight. In another report, Patel et al analyzed 2133 US children who underwent HT for dilated cardiomyopathy during 1989–2012 (2). DR size mismatch using either weight or height was not associated with post-transplant survival in multivariable analysis. The 2019 annual ISHLT pediatric analysis did not find association of DR weight mismatch with 1 year post-transplant mortality in adjusted analysis (*p* = .09) (3). The association of using hearts from undersized donors with post-transplant in-hospital mortality in the current analysis illustrates that the major consideration in DR size match is limited to the immediate post-transplant period. The loss of this association with longer follow-up may be explained by echocardiographic studies in pediatric HT recipients with DR size mismatch that have shown that LV mass regresses or grows to become near-normal for the recipient size within the first few weeks and months post-transplant (19, 20). Furthermore, the number of recipients exposed to this risk factor was small (one 10th of the cohort). Therefore, when analyzed for the full cohort, the risk was short-lived, and with time, other factors that were important in the full cohort became more important.

### Study Implications

Our analysis shows a significant association of HT from undersized donors with early post-transplant mortality. Because cardiac mass in normal children increases as the body size increases (8), the association when expressed as donor-recipient PLM ratio - while performing similar to the DR body size ratios—provides a physiologic correlate for the risk associated with undersized donors. If the ultimate goal is to match the donor and the recipient for their predicted heart mass, the weight ratio and BSA ratio appear to be reasonable surrogates in children unlike in adults. The difference between adults and children in this regard may be best explained by the gender difference in calculation of predicted heart mass. It is notable that the pediatric LV mass equation is the same in boys and girls with similar BSA. In contrast, there is a significant difference in values for the predicted LV (and RV) mass by gender such that with the same body weight and height as that of a man, the LV mass in a woman calculates to 82.7% of that man, thus explaining the increased risk of mortality in adult male recipients when receiving HT from a female donor. This is the likely explanation for a much superior performance of heart mass calculation in adult HT recipients over body measurements whereas they appear to perform no differently in children.

The size match categories in our analysis were chosen to understand if either undersized or oversized donors were associated with worse outcomes compared to the reference group and were guided in part by an adult study where seven equal-size groups were analyzed for size match with just the middle group being the reference group (6). With a much smaller study population in children, we needed the reference group to be larger than the exposed groups. Because PLM ratio is a continuous variable, we also analyzed it as such and as expected, the risk of graft loss was higher the more undersized the donor. Our primary study finding does indeed support the current clinical practice of caution with undersized donors and defines the threshold to be donor-recipient PLM ratio of ≤.9 or weight ratio <.88 or BSA ratio of <.92, each seen in pediatric HT in 10% of all recipients. It is important to note however, that despite the higher relative risk, the observed outcomes seen with such undersized donor hearts may be considered quite reasonable in many HT candidates who may not otherwise receive another donor call. The decision when evaluating such donors would require one to balance the consequences of accepting an undersized heart vs the risk of wait-list mortality.

### Limitations

This study has several limitations. First, this was a retrospective study using registry data with inherent limitations of such data. However, submission of these data to UNOS by centers is required, the data are used on an ongoing basis for organ allocation and are periodically audited by UNOS, thus allowing safeguards to data quality. Second, although we describe increased mortality risk in 10% of HT recipients with the most undersized donors, the category as defined is somewhat arbitrary and the risk is continuous with a higher risk the more undersized the donor rather than present at a specific PLM ratio. Third, donor-recipient size mismatch may be clinically reasonable in a cachectic or an overweight recipient in whom ideal body weight, such as the 50th percentile weight for current height, instead of the current weight, may be considered. We did not analyze such examples in this study for statistical reasons.

## Conclusion

Pediatric HT recipients who receive hearts from donors with donor-recipient PLM ratio ≤.9 are at significantly increased risk of early post-transplant mortality. However, this metric is not superior to donor-recipient weight ratio or BSA ratio when assessing size match as this association is also seen when evaluating donors and recipients using weight ratio or BSA ratio. These findings should be considered during decision making when assessing potential donors for HT candidates.

## Capsule Sentence Summary

A longstanding wisdom when evaluating a donor heart for a heart transplant candidate is to avoid undersized donors due to the risk of primary graft failure. However, previous analyses in pediatric heart transplant recipients using weight or height have not found donor-recipient size-mismatch to be associated with post-transplant mortality. A recent study in healthy US children using echocardiography described an equation for LV mass using body surface area. We assessed if donor-recipient size mismatch assessed using predicted LV mass ratio is associated with post-transplant mortality. In a study of 4,717 pediatric heart transplants in the US over 15 years study duration, we found that children with donor-recipient predicted LV mass ratio <.9 (10% with most undersized donor hearts) were at higher risk of post-transplant in-hospital mortality adjusted for other risk factors. The metric was not superior to donor-recipient weight ratio or BSA ratio for assessing size match however because recipients in the lowest decile of donor-recipient weight ratio or body surface area ratio were also at increased risk of in-hospital mortality.

## Data Availability

The data analyzed in this study is subject to the following licenses/restrictions: The data are available upon request to participating centers in the US. Requests to access these datasets should be directed to https://optn.transplant.hrsa.gov › data › request-data.

## Abbreviations

BSA, body surface area; CI, confidence interval; DR, donor-recipient; GFR, glomerular filtration rate; HR, hazard ratio; HT, heart transplant; LV, left ventricle; MRI, magnetic resonance imaging; OR, odds ratio; OPTN, Organ Procurement and Transplantation Network; PLM, predicted LV mass ratio; RV, right ventricle; US, United States.
