# Supplementary material for: Matching Donor and Recipient Size in Pediatric Heart Transplantation
Source: Transpl Int. 2022 Feb 7;35:10226. doi: 10.3389/ti.2022.10226 (PMC8842272; doi:10.3389/ti.2022.10226)
Supplement: Supplementary file 1 [file DataSheet1.docx]

**Supplementary Digital Content**

**Table S1: Risk for Post-Transplant in-hospital Mortality, by Group**

|  | **Unadjusted** | | | **Adjusted*** | | |
| --- | --- | --- | --- | --- | --- | --- |
|  | **OR** | **95% CI** | **P Value** | **OR** | **95% CI** | **P Value** |
|  |  |  |  |  |  |  |
| **PLM Ratio Group** |  |  |  |  |  |  |
| 1 | 1.54 | 1.07, 2.22 | 0.020 | 1.55 | 1.04, 2.32 | 0.031 |
| 2 | 0.88 | 0.56, 1.38 | 0.57 | 1.01 | 0.62, 1.64 | 0.97 |
| 3 | 1.00 | -- | -- | 1.00 | -- | -- |
| 4 | 1.29 | 0.87, 1.90 | 0.20 | 0.95 | 0.62, 1.46 | 0.82 |
| 5 | 1.25 | 0.84, 1.85 | 0.27 | 0.78 | 0.50, 1.20 | 0.26 |
| **Weight Ratio Group** |  |  |  |  |  |  |
| 1 | 1.44 | 1.00, 2.08 | 0.051 | 1.49 | 0.99, 2.25 | 0.054 |
| 2 | 0.62 | 0.37, 1.02 | 0.062 | 0.79 | 0.47, 1.35 | 0.39 |
| 3 | 1.00 | -- | -- | 1.00 | -- | -- |
| 4 | 1.24 | 0.84, 1.82 | 0.28 | 1.07 | 0.70, 1.63 | 0.75 |
| 5 | 1.24 | 0.84, 1.82 | 0.28 | 0.72 | 0.47, 1.11 | 0.13 |
| **Height Ratio Group** |  |  |  |  |  |  |
| 1 | 1.11 | 0.74, 1.68 | 0.60 | 1.15 | 0.74, 1.77 | 0.54 |
| 2 | 1.20 | 0.80, 1.78 | 0.38 | 1.38 | 0.89, 2.14 | 0.15 |
| 3 | 1.00 | -- | -- | 1.00 | -- | -- |
| 4 | 1.32 | 0.90, 1.94 | 0.16 | 0.99 | 0.65, 1.51 | 0.96 |
| 5 | 1.24 | 0.84, 1.83 | 0.29 | 0.73 | 0.47, 1.13 | 0.16 |
| **BSA Ratio Group** |  |  |  |  |  |  |
| 1 | 1.50 | 1.03, 2.18 | 0.034 | 1.53 | 1.02, 2.30 | 0.042 |
| 2 | 0.94 | 0.61, 1.47 | 0.80 | 1.17 | 0.73, 1.89 | 0.52 |
| 3 | 1.00 | -- | -- | 1.00 | -- | -- |
| 4 | 1.54 | 1.07, 2.23 | 0.022 | 1.19 | 0.79, 1.79 | 0.41 |
| 5 | 1.28 | 0.86, 1.90 | 0.22 | 0.78 | 0.50, 1.20 | 0.26 |
|  |  |  |  |  |  |  |

* Odds ratios are adjusted for all variables in the multivariable model for in-hospital mortality (Table 2)

PLM = predicted LV mass, BSA = body surface area

**Table S2: Risk for Graft Loss within 1 Year of Heart Transplant, by Group**

|  | **Unadjusted** | | | **Adjusted*** | | |
| --- | --- | --- | --- | --- | --- | --- |
|  | **HR** | **95% CI** | **P Value** | **HR** | **95% CI** | **P Value** |
|  |  |  |  |  |  |  |
| **PLM Ratio Group** |  |  |  |  |  |  |
| 1 | 1.18 | 0.88, 1.58 | 0.26 | 1.20 | 0.89, 1.61 | 0.23 |
| 2 | 0.92 | 0.67, 1.27 | 0.61 | 1.03 | 0.74, 1.42 | 0.87 |
| 3 | 1.00 | -- | -- | 1.00 | -- | -- |
| 4 | 1.13 | 0.84, 1.52 | 0.42 | 0.91 | 0.67, 1.22 | 0.52 |
| 5 | 1.37 | 1.04, 1.80 | 0.023 | 1.00 | 0.75, 1.32 | 0.98 |
| **Weight Ratio Group** |  |  |  |  |  |  |
| 1 | 1.06 | 0.79, 1.43 | 0.70 | 1.10 | 0.81, 1.48 | 0.54 |
| 2 | 0.73 | 0.52, 1.04 | 0.079 | 0.91 | 0.65, 1.30 | 0.62 |
| 3 | 1.00 | -- | -- | 1.00 | -- | -- |
| 4 | 1.09 | 0.81, 1.46 | 0.59 | 0.98 | 0.72, 1.32 | 0.87 |
| 5 | 1.27 | 0.96, 1.67 | 0.089 | 0.88 | 0.67, 1.17 | 0.39 |
| **Height Ratio Group** |  |  |  |  |  |  |
| 1 | 1.26 | 0.94, 1.68 | 0.12 | 1.23 | 0.92, 1.64 | 0.16 |
| 2 | 0.95 | 0.69, 1.32 | 0.77 | 1.11 | 0.81, 1.54 | 0.51 |
| 3 | 1.00 | -- | -- | 1.00 | -- | -- |
| 4 | 1.24 | 0.93, 1.66 | 0.15 | 1.02 | 0.76, 1.37 | 0.91 |
| 5 | 1.53 | 1.17, 1.99 | 0.002 | 1.03 | 0.79, 1.36 | 0.81 |
| **BSA Ratio Group** |  |  |  |  |  |  |
| 1 | 1.11 | 0.82, 1.50 | 0.49 | 1.14 | 0.85, 1.55 | 0.38 |
| 2 | 0.87 | 0.63, 1.21 | 0.40 | 1.03 | 0.74, 1.44 | 0.86 |
| 3 | 1.00 | -- | -- | 1.00 | -- | -- |
| 4 | 1.29 | 0.97, 1.70 | 0.080 | 1.04 | 0.79, 1.39 | 0.77 |
| 5 | 1.33 | 1.01, 1.75 | 0.045 | 0.95 | 0.72, 1.26 | 0.73 |
|  |  |  |  |  |  |  |

* Hazard ratios are adjusted for all variables in the Cox regression multivariable model for 1-year graft loss (Table 3)

PLM = predicted LV mass, BSA = body surface area
